# *Acinetobacter baumannii* Virulence Traits: A Comparative Study of a Novel Sequence Type with Other Italian Endemic International Clones

**DOI:** 10.3389/fmicb.2017.01977

**Published:** 2017-10-12

**Authors:** Cecilia Ambrosi, Daniela Scribano, Marta Aleandri, Carlo Zagaglia, Laura Di Francesco, Lorenza Putignani, Anna Teresa Palamara

**Affiliations:** ^1^Department of Public Health and Infectious Diseases, Sapienza University of Rome, Rome, Italy; ^2^Dani Di Giò Foundation–Onlus, Rome, Italy; ^3^Department of Medical, Oral and Biotechnological Sciences, Università degli Studi “G. d’Annunzio” Chieti–Pescara, Chieti, Italy; ^4^Unit of Human Microbiome, Bambino Gesù Children’s Hospital, IRCCS, Rome, Italy; ^5^Unit of Parasitology, Bambino Gesù Children’s Hospital, IRCCS, Rome, Italy; ^6^Department of Public Health and Infectious Diseases, Sapienza University of Rome, Laboratory Affiliated to Institute Pasteur Italia, Cenci-Bolognetti Foundation, Rome, Italy; ^7^San Raffaele Pisana, IRCCS, Telematic University, Rome, Italy

**Keywords:** *Acinetobacter baumannii*, motility, biofilm, oxacillinases, host-pathogen interactions

## Abstract

Carbapenem-resistant *Acinetobacter baumannii* (CRAb) have emerged in recent decades as major causes of nosocomial infections. Resistance is mainly due to overexpression of intrinsic and/or acquired carbapenemases, especially oxacillinases (OXA). In Italy, although the sequence type (ST) 2 and the ST78 are the most frequently detected, we recently reported ST632, a single locus variant of ST2. Therefore, this study was aimed at unraveling common bacterial surface virulence factors involved in pathogenesis and antibiotic resistance in representative CRAb of these ST genotypes. Outer membrane protein (OMP) composition together with motility, biofilm formation, *in vitro* adherence to, invasion of, and survival within pneumocytes were analyzed. Differently from the carbapenem-susceptible reference strain ATCC 17978, either overexpressed OXA-51 or both OXA-23 and OXA-51 co-purified with OMPs in CRAb. This tight association ensures their maximal concentration on the inner surface of the outer membrane to provide the best protection against carbapenems. These findings led us to propose for the first time a common behavior of OXA enzymes in CRAb. Despite the presence of both OmpA and phosphorylcholine-porinD and the ability of all the strains to adhere to cells, invasion, and survival within pneumocytes was shown only by ST2 and ST78 isolates, sharing the highest number of identified OMPs. Conversely, notwithstanding genetic and OMPs similarities with ST2, ST632 was unable to invade and survive within epithelial cells. Overall, our study shows that different STs share a specific OMP composition, also shaped by overexpressed OXA, that is needed for invasiveness and survival of CRAb.

## Introduction

*Acinetobacter baumannii* is an opportunistic Gram-negative pathogen that has emerged in recent decades as a worldwide cause of nosocomial infections associated with elevated morbidity and mortality (Wong et al., 2017). The major concern of *A. baumannii* infections is that clinical isolates of this organism are often resistant to multiple types of antimicrobial therapies (Wong et al., 2017). Initially, carbapenems were used to treat *A. baumannii* infections; however, carbapenem-resistance *A. baumannii* (CRAb) increased significantly among clinical isolates (Wong et al., 2017). At present, treatment options for *A. baumannii* Extensively Drug-Resistant (XDR) strains are increasingly limited (Wong et al., 2017). Both non-enzymatic and enzymatic mechanisms of carbapenem resistance have been described in CRAb (Nowak and Paluchowska, 2016). The non-enzymatic mechanism relies on the upregulation of the three efflux systems, alterations of target penicillin-binding proteins, and changes in the outer membrane protein (OMP) composition (Nowak and Paluchowska, 2016). Instead, the enzymatic carbapenem-resistance mechanism depends on the overexpression or derepression of β-lactamases belonging to Ambler classes A, B, and D of β-lactamases which break the amide bond of the β-lactam ring using different hydrolytic mechanisms of antibiotic inactivation (Nowak and Paluchowska, 2016). Noteworthy, carbapenem-hydrolyzing class D β-lactamases (CHDLs), also known as oxacillinases (OXA), are the most widely β-lactamases found in *A. baumannii* clinical isolates. Six different OXA groups have been identified, OXA-51-like, OXA-23-like, OXA-40/24-like, OXA-58-like, OXA-143-like, and OXA-48-like (Nowak and Paluchowska, 2016). The intrinsic chromosomal *bla*_OXA-51-like_ alleles encoding more than 95 OXA-51-like variants are naturally found in the chromosome of all *A. baumannii* strains; however, overexpression of the OXA enzyme conferring carbapenem resistance occurs through insertion of sequence elements upstream the *bla*_OXA_ genes (Nowak and Paluchowska, 2016). Interestingly, the global distribution of CRAb was found to be associated to diverse genetic backgrounds, although predominating strains can be grouped into clonal complexes (Karah et al., 2012). Indeed, epidemiological studies based on multilocus sequence typing (MLST) demonstrated the occurrence of three major clones as responsible for outbreaks in Europe (European clones 1–3) and thereafter, worldwide, imposing to rename them as International Clones (ICs) (Diancourt et al., 2010). Currently, 18 ICs have been found globally distributed, two restricted to Asia and six to Europe (Karah et al., 2012). Apart from strains of sequence type (ST) 2 (Pasteur’s MLST scheme), belonging to the very successful IC 2, outbreaks in Italy are mainly caused by ST78 (Pasteur’s MLST scheme) known as the “Italian clone,” belonging to IC 6 (Diancourt et al., 2010; Giannouli et al., 2010, 2013; Karah et al., 2012; Principe et al., 2014). Using the same MLST scheme, we have previously shown the perpetration of both ST2 and ST78 strains in an Italian intensive care unit (ICU) and the advent of the new ST632 for the first time in Italy, representing a single locus variant (within the *rpoB* allele) of the widespread ST2 (Ambrosi et al., 2016). These CRAb showed also an XDR antibiotype, being susceptible only to colistin (Ambrosi et al., 2016).

Although the huge number of studies reporting on molecular epidemiology and antimicrobial resistance profiles of *A. baumannii* clinical isolates (Diancourt et al., 2010; Zarrilli et al., 2013; Potron et al., 2015), data accounting on different virulence factors of individual strains are limited. Several studies reported on major differences in virulence-associated traits in *A. baumannii* isolates, such as biofilm formation, adherence to human epithelial cells, invasion, motility, and cytotoxicity (Choi et al., 2008a; Smani et al., 2011, 2013; Weber et al., 2015; Vijayakumar et al., 2016). These bacterial features mostly depend on bacterial cell surface constituents and are often intimately interconnected. An important group of proteins that can affect the virulence of different isolates is that of the OMPs. To this group belongs the well known OmpA protein which has been shown to be implicated in adherence to and invasion of epithelial cells, in biofilm-forming activity, in antimicrobial resistance, and cell death, as well as the Omp33-36 (also known as 34 kDa) (Choi et al., 2008a,b; Gaddy et al., 2009; Smani et al., 2013). Likewise, porinD, an OMP belonging to the OprD family, containing the small molecule phosphorylcholine, was shown to be involved in bacterial adherence/invasion of eukaryotic cells and carbapenem resistance (Smani et al., 2012; Smani and Pachón, 2013). Furthermore, the two-partner secretion system (TPS), FhaB/FhaC, was shown to be involved in mediating tight adherence to eukaryotic cells (Pérez et al., 2016).

Therefore, the aim of the present work was to use a wide approach to examine cell surface virulence factors of STs commonly isolated in Italian hospitals (i.e., ST2 and ST78) and the newly identified ST632 involved in their ability to colonize biotic and abiotic surfaces and antibiotic resistance. OMP profiles, motility, biofilm formation, adherence to, invasion of, and survival within human lung epithelial cells were compared among STs and the ATCC 17978 reference strain. Data presented herein demonstrate for the first time that the presence of both OmpA and phosphorylcholine-porinD is necessary but not sufficient for host cell invasion. Additional OMPs, shared by different STs, are needed for CRAb invasiveness and survival within the host cells. This OMP composition is further influenced by overexpressed OXA enzymes that directly interact with OMPs for functional localization.

## Materials and Methods

### Bacterial Strains and Growth Conditions

Three representative CRAb, belonging to the most common Italian STs and to a new ST, were selected from a previous study on 31 *A. baumannii* strains isolated from respiratory specimens of patients admitted to the ICU at the University Hospital Policlinico Umberto I of Rome, Italy (Ambrosi et al., 2016). Selected isolates were all XDR strains, yet still susceptible to colistin. The carbapenem-susceptible ATCC17978 reference strain was used as control. The main characteristics of *A. baumannii* strains are listed in Supplementary Table S1. Bacteria were grown in Luria-Bertani (LB) or Brain Hearth Infusion (BHI) broth (Difco, Italy). The growth kinetic of each isolate was determined at 37°C in LB broth with vigorous shaking (200 rpm). Cell densities (OD_600_) and colony-forming units (CFU) were determined every hour over a 5-h period.

### Motility Assay

Surface motility was investigated as previously described (Clemmer et al., 2011). Agar (Difco) was added to a final concentration of 0.25%. Plates were incubated at 37°C for 16 h, and the growth area (cm^2^) was measured. Three independent experiments in duplicate were performed.

### Biofilm Assay

Biofilm formation was measured using the microtiter plate assay (Stepanović et al., 2007). Briefly, overnight cultures were diluted 1:50 in 200 μl of LB and dispensed into 96-well polystyrene microtiter plates (Costar, Corning Inc.) and incubated at 37°C for 24 h under static conditions. Following OD_600_ measurements, plates were washed three times with phosphate-buffer saline solution (PBS), fixed with methanol for 20 min at room temperature, and stained with 0.1% crystal violet solution for 15 min. After four additional washes with water, the surface-associated dye was solubilized with 200 μl of 95% ethanol and OD_570_ was recorded. Results are reported as the OD_570_/OD_600_ ratio to normalize the amount of biofilm formed to the total bacterial content. Three independent experiments, eight wells per strain, were performed. Isolates were classified as biofilm-forming if they yielded ratio values that were at least three standard deviations above that of uninoculated medium (0.22), considered as the negative control (Stepanović et al., 2007).

### Sodium Dodecyl Sulfate Polyacrylamide Gel Electrophoresis (SDS-PAGE), Western Blot Analysis and In-Gel Digestion

Whole cell extracts (WCEs) were prepared by lysing exponentially grown bacteria in 1X Laemmli buffer (Laemmli, 1970). In parallel, the same bacterial cultures were washed twice with PBS5 and OMPs were extracted as described previously (Cuenca et al., 2003). Briefly, bacterial cells were sonicated, treated with *N*-lauryl-sarcosine and resuspended in Cracking Dye (2% SDS, 20% glycerol, 62.5 mM Tris-HCl pH 6.8, 0.05% bromophenol blue, and 5% β-mercaptoethanol); WCEs were denatured for 10 min whereas OMPs for 5 min. Proteins were resolved by 12.5% Tris-glycine sodium dodecyl sulfate polyacrylamide gel electrophoresis (SDS-PAGE), and stained with Coomassie brilliant blue R-250 (Sigma) or electrotransferred onto PVDF membranes (Hybond-P, Millipore). Blots were probed with *A. baumannii* anti-OmpA serum (a kind gift of Prof. Smani) and with anti-phosphorylcholine (ChoP) TEPC 15 monoclonal antibody (Sigma), and an anti-mouse secondary antibodies IgG and IgA conjugated to horseradish peroxidase, respectively (Bio-Rad). Blots were visualized by enhanced chemiluminescence system (GE-Healthcare Bio-Sciences). From the Coomassie-stained SDS-PAGE, four gel slices were excised from each lane and subjected to in-gel digestion with trypsin, as previously described (Di Francesco et al., 2012). The tryptic peptides were extracted and dried in a speed vacuum.

### LC-MS/MS Analysis

The peptide mixtures were analyzed by a 5600+ TripleTOF mass spectrometer (AB SCIEX, Canada), which was equipped with a nanoelectrospray ion source. The tryptic peptides were automatically loaded into an Eksigent Ekspert NanoLC 400 system (AB SCIEX) and desalted on a C18 trap column (2 cm, ID 100 μm, 5 μm). The peptide mixtures were separated with a constant flow of 300 nL/min. on a C18 analytical column (25 cm, ID 75 μm, 5 μm) at a temperature of 40°C by a two-step gradient of solvent B (98% acetonitrile with 0.1% formic acid). Mass spectral data were acquired in a positive mode into the TripleTOF operating in information-dependent acquisition (IDA) mode. Survey TOF MS scans from 350–1250 *m/* were acquired in 0.25 s. MS/MS analysis through collision-induced dissociation (CID) was performed on the 35 most intense ions with charge states 2^+^–5^+^ detected per survey scan if they exceeded an intensity of at least 70 counts/s. For MS/MS scanning, the accumulation time was set to 0.1 s from 230 to 1500 *m/z*. To avoid redundant sequencing of the most abundant peptides, the active exclusion was enabled for 30 s after two MS/MS scans on the same precursor ion.

### Database Search and Protein Identification

The acquired spectra were loaded to the ProteinPilot v. 4.5 (AB SCIEX) search program and the Paragon algorithm was used to search peak lists against *A. baumannii* database from UniProtKB (release 2017-01-31), containing 452,517 protein entries. In the search parameters, trypsin was used as the proteolytic enzyme and the number of allowed missed cleavages was two. Oxidation (methionine) was used as variable modification, carbamidomethylation (cysteine) as fixed modification and biological modifications programmed in algorithm were permitted. All settings for mass tolerance were as default. A false discovery rate (FDR) analysis of the results has been carried out and only proteins identified at 1% global FDR on the reverse sequence of the protein sequence FASTA file have been selected. To minimize false positive results, a strict cut-off for protein identification was applied with a number of distinct peptides having at least 95% confidence >3 and the unused protein score ≥1.3, which corresponds to a confidence limit of 95%.

Functional annotation analysis of identified proteins was performed using the comprehensive bioinformatics tool UniProtKB.^1^ Subcellular localization was also predicted using both PSORTII and PSORTb tool v. 3.0.2.^2^ The Venn diagram of identified proteins was generated using the graphic tool Venny 2.1.0.^3^

### Adherence, Invasion, and Survival Assays

The human A549 lung epithelial cell type II line (ATCC CCL185) was cultured in Dulbecco’s modified Eagle’s medium (DMEM) supplemented with 10% fetal bovine serum (FBS) and grown in the presence of 5% CO_2_ at 37°C. Semi-confluent cell monolayers were infected at a multiplicity of infection (MOI) of 100, centrifuged, incubated for 60 min at 37°C in 5% CO_2_, and washed five times with PBS before being lysed with 0.1% Triton X-100. Serially diluted lysates were plated on LB agar plates to determine the number of adherent bacteria (CFU/ml). At the same time, the medium overlaying monolayers was replaced with fresh culture medium containing 5 μg/ml of colistin sulfate (BioChemica) to kill extracellular bacteria and incubated for further 12, 24, and 48 hrs at 37°C in 5% CO_2_. At each time point, cells were washed three times with PBS and lysed with 0.1% Triton X-100. Undiluted and serially diluted lysates were plated on LB agar plates to determine invading and surviving bacteria (CFU/ml). Following each infection steps, from invasion to survival, bacteria were subcultured in the presence of colistin both in LB broth and plates in order to rule out the onset of colistin-resistant bacteria. Invasion was considered to be significant if a minimum of ≥0.3% of the initial inoculum could be recovered from intracellular compartments.

### Fluorescence Microscopy

Infected A549 cell monolayers were washed with PBS, fixed with 4% paraformaldehyde and permeabilized for 10 min with a 0.25% solution of Triton X-100 in PBS. Bacterial and cellular DNAs were labeled with 4′,6′-diamidino-2-phenylindole (DAPI, Molecular Probes), whereas F-actin structures were visualized with rhodamine-conjugated phalloidin (Sigma). Cell morphology and chromatin condensation of infected and uninfected cells were evaluated by phase contrast and fluorescence microscopy, respectively. Analysis and count were evaluated in 10^4^ cells per sample in at least three separate experiments. Single images were acquired with a Leica DM5000B microscope equipped with the Digital FireWire Color and Black and White Camera systems LeicaDFX350 and DFX300, respectively, and processed using the Leica Application Suite 2.7.0.R1 software (Leica).

### PCR Detection of the *fhaC* Gene

*Acinetobacter baumannii* templates were prepared from a single colony grown on LB agar plates and resuspended in PCR grade water. The sense primers FWtRNA (5′ GGATTGGTGTAATGGTAGCAT 3′), FWfhaCINT (5′ TGCACATTTGTGGAATGCTTTA 3′), and FWfhaCINT2 (5′ ATAAGATTTCAGCTCAGC 3′) were used in combination with the anti-sense primer RVfhaC (5′ AAGTTCTGGACCTTTAAACTTG 3′) to amplify the full-length and internal regions of the *fhaC* gene. The PCR was performed using the DreamTaq PCR Master Mix, following manufacturers’ instructions (Thermo Fisher Scientific Inc.) and a Tm of 58°C. Amplicons were purified using the PCR Clean-up kit (Macherey-Nagel) and sequenced (BioFab).

### Nucleotide Sequence Accession Number

The sequence of the *fhaC* gene found in the isolate #36 (ST78) has been deposited in GenBank database under the accession no. KY593933.

### Statistical Analyses

The statistical differences were analyzed by one-way ANOVA and *post hoc* Student’s *t*-test. Values of *P* < 0.05 were taken as being statistically significant.

## Results

### Acquirement of Antibiotic-Resistance Did Not Alter Growth Rates in *A. baumannii* Isolates

*Acinetobacter baumannii* clinical isolates selected for this study were representative of different clonal populations isolated and characterized previously (Ambrosi et al., 2016). The main characteristics of isolates are summarized in Supplementary Table S1. Since it has been reported that acquisition of antibiotic resistance mechanisms might have an impact in bacterial fitness due to its high biological cost (Beceiro et al., 2013), *in vitro* growth curves of all isolates were compared. No significant difference in the growth rates among isolates and the ATCC 17978 reference strain was found (*P* > 0.05; data not shown).

### Acquirement of Antibiotic Resistance Was Shown to Be Associated with Strain-Specific Differences of the OMP Profile

**FIGURE 1:**
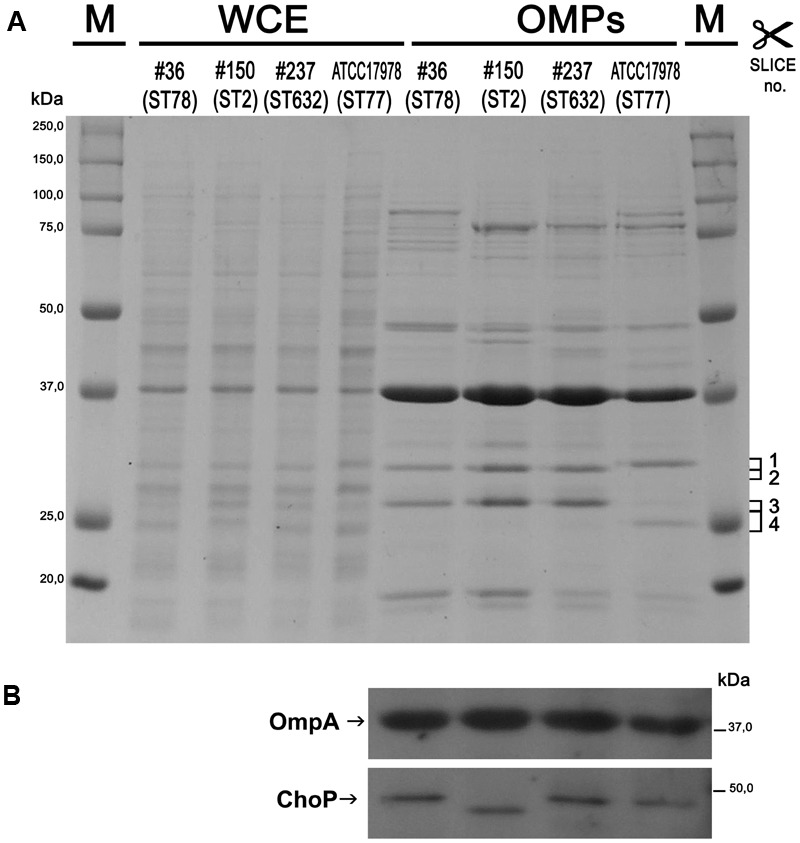
Protein profiles and analysis of the outer membrane (OM) fractions from carbapenem-resistant *Acinetobacter baumannii* (CRAb). Proteins present in WCEs and in the outer membrane protein (OMP) fractions were stained with Coomassie brilliant blue **(A)** or transferred to a PVDF membrane and probed using anti-OmpA and anti-ChoP TEPC 15 antibodies **(B)**. Size markers (kDa), the four gel slices excised and subjected to in-gel digestion (complete data are presented in Supplementary Table S2) and the positions and names of OmpA and ChoP are indicated.

The outer membranes (OM) of Gram-negative bacteria contain a variety of β-barrel OMPs necessary for cell survival, pathogenesis, and adaptation in host cells as well as antibiotic resistance. Therefore, the pattern of WCEs and purified OMPs was compared on SDS-PAGE by Coomassie blue staining. Major differences were observed in OMP banding patterns among isolates, particularly for high molecular weight proteins (**Figure 1A**). A protein band of the expected size for OmpA (38 kDa) was easily detectable in the WCEs of each isolate, and its intensity increased especially in the OMP fraction (**Figure 1A**). The identity of the OmpA protein was further confirmed by Western blot analysis using *A. baumannii* anti-OmpA antibody (**Figure 1B**). Detection of the OMP porinD bound to phosphorylcholine was performed using anti-ChoP specific antibody, as previously described (Smani et al., 2012). A band of 43 kDa was highly detectable in the OMP fraction of all strains; however, the corresponding band in isolate #150 (ST2) displayed a lower molecular weight in comparison to the other strains (**Figure 1B**).

**FIGURE 2:**
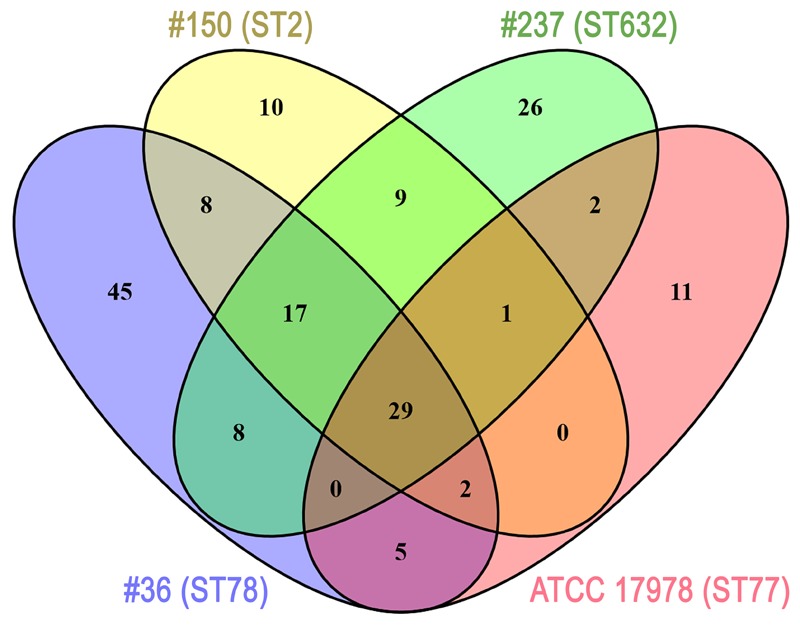
Venn diagram showing the number of proteins shared among *A. baumannii* strains. Each *A. baumannii* isolate is depicted by a different oval color, as indicated. Proteins included were those identified by MS (cut-off: protein confidence = 95%) in the 20–36 kDa range from the Coomassie-stained gel shown in **Figure 1**, and having at least 95% = 4 minimum peptides. Numbers in the non-overlapping regions show the number of proteins unique to that strain. Supplementary Table S2 contains the information shown in this diagram at the protein level.

Several differences were observed in the OMP profiles between the ATCC 17978 reference and CRAb isolates, especially for OMPs in the range of 25–33 kDa (**Figure 1A**). Hypothesizing that proteins highly detectable only in CRAb might be related to antimicrobial resistance, four SDS-PAGE gel slices were excised from the OMP fraction of each strain, subjected to in-gel tryptic digestion, and extracted peptides were subjected to LC-MS/MS analysis. For protein identification, MS data were matched against *A. baumannii* database from UniProtKB; a total of 183 proteins were confidently identified (Supplementary Table S2). Similarly to previous reports on OMP fractions recovered using different purification methods (Thein et al., 2010 and references therein), several cytoplasmic proteins, mostly ribosomal proteins, were retrieved. Nevertheless, one third of identified proteins appeared to possess a signal peptide and to be located within the OM (**Table 1**). The Venn diagram was used to evaluate the number of proteins shared among different *A. baumannii* strains (**Figure 2**). Indeed, taking into account the proteins identified with a 95% confidence, 27 proteins were found in all *A. baumannii* strains, 15 proteins in CRAb exclusively, and 14 proteins only in the ATCC 17978 reference strain (**Figure 2** and Supplementary Table S2). Next, to identify differentially expressed proteins among CRAb and ATCC 17978 reference strain, in the range of 20–36 kDa, a further analysis was performed (**Table 1**). Highly detectable OMPs were CarO, YiaD, Omp33–36 kDa, lipoproteins, and conjugative Tra proteins (**Table 1**). Both CarO and Omp33–36 kDa are porins known to participate in the influx of carbapenems (Limansky et al., 2002; del Mar Tomás et al., 2005). Interestingly, the Omp33–36 kDa porin was strongly expressed in all strains analyzed (Supplementary Table S2). Conversely, CarO was found to be significantly expressed in all CRAb, whereas below our cut-off value in the ATCC 17978 reference strain (**Table 1**).

**Table 1:** Identification of differentially expressed proteins from the OM fractions of carbapenem-resistant *Acinetobacter baumannii* (CRAb) and ATCC 17978 reference strain, matching criteria described in Section “Materials and Methods” and with a MW included in the range of 20–36 kDa.

			Peptides (95%)^2^				
Accession	Protein	Biological	#36	#150	#237	ATCC 17978	Localization/secretion
code^1^	name	process	(ST78)	(ST2)	(ST632)	(ST77)	prediction^3^
Q155P8	OM protein CarO	Transport of molecules across the OM	640	661	361		OM/Yes
D7RPF0	Carbapenem-hydrolyzing oxacillinase OXA-176 (OXA-51-like protein)	Penicillin binding	16	28	5		PP/Yes
U1TWU9	Membrane protein	Unknown	7	6	4		IM/Yes
V5V9C7	Protein-export membrane protein SecF	Intracellular protein transmembrane transport	6	6	4		IM/No
A0A1E3M8B2	NLPA lipoprotein	Unknown	28			20	IM/Yes
V5VAD3	Succinate dehydrogenase SdhB	Oxidation-reduction	24	12		12	IM/No
V5VGI9	DNA-binding protein	DNA-binding	18			17	IM/No
U1W0D3	Ion channel protein Tsx	Unknown	10			7	OM/Yes
V5V7X6	OM family protein	Unknown	9	5			OM/Yes
V5VEU8	Metalloprotease LoiP	Proteolysis	9	4		4	Unknown/Yes
A0A009KJ91	Gram-negative pili assembly chaperone, N-terminal domain protein	Cell wall organization	8	36			OM-bound PP/Yes
U1UE18	PaaX-like protein C-terminal domain protein	Unknown	7				Unknown/No
U1VKW0	Membrane protein	Unknown	6		4		IM/Yes
A0A062ETJ9	Copper resistance protein CopB	Cellular copper ion homeostasis	6				OM/Yes
S3T2J4	Beta-hydroxylase	Peptidyl-amino acid modification	5				IM/No
V5VG76	OM lipoprotein omp16	Transport of molecules across the OM	4	4		5	OM/Yes
V5VH63	Bacterial type II secretion system protein N	Unknown	4				IM/No
U1UED1	General secretion pathway protein	Intracellular protein transmembrane transport	4				IM/No
V5VJ04	Signal peptide protein	Unknown	4				OM/Yes
Q9L4P2	Beta-lactamase OXA-23	Penicillin binding		84	36		PP/Yes
U5QES9	Membrane lipoprotein lipid attachment site	Unknown		7			Unknown/Yes
U5QEZ8	Conjugative transfer system protein TraK	Unknown		6			Unknown/Yes
U1TMC1	5′-nucleosidase	Nucleoside metabolism			4		Unknown/No
G2JDI0	Alpha-beta hydrolase family esterase	Lipid metabolism			4		Unknown/No
U1UBC5	Aspartyl/asparaginyl beta-hydroxylase	Peptidyl-amino acid modification			4		IM/No
G2JD82	Lipoprotein-releasing system ATP-binding protein LolD	Lipoprotein transport			4		IM/No
A0A1E3M6L8	FHA domain-containing protein	Unknown				8	IM/No
A0A0B2XZJ2	Membrane protein	Unknown				4	IM/No
A0A009GZE7	Metallo-beta-lactamase superfamily protein	Pyruvate metabolism				4	Unknown/No
V5VG30	NADH-quinone oxidoreductase subunit B	Oxidation-reduction		4		4	IM/No

*^1^UniProt accession number. ^2^The number of distinct peptides having at least 95% confidence. ^3^The presence of the signal peptides and the subcellular localization were predicted using both PSORT II and PSORTb (version 3.0.2) or based on literature data. Differentially expressed proteins annotated as cytosolic were omitted. OM, outer membrane; IM, inner membrane, PP, periplasm.*

Our previous genetic screening showed that both #150 (ST2) and #237 (ST632) isolates contained both the intrinsic *bla*_OXA-51_ gene and an acquired allele encoding an OXA-23-like enzyme, whereas #36 (ST78) isolate had the *bla*_OXA-51-like_ as the only carbapenemase gene to confer resistance to carbapenems (Ambrosi et al., 2016). As expected, the sole intrinsic *bla*_OXA-51-like_ allele was detected in the carbapenem-susceptible ATCC 17978 reference strain (Ambrosi et al., 2016). Remarkably, we found OXA β-lactamases belonging to the OXA-23-like and/or OXA-51-like groups in the OMP fractions of CRAb, whereas no OXA-51-like enzyme could be detected in the OM fraction from ATCC 17978 reference strain (**Table 1**). While co-purification of OXA-23-like enzymes within the OMP fraction was previously reported (Fajardo Bonin et al., 2014; Schweppe et al., 2015; Wu et al., 2016), to our knowledge, this is the first study to demonstrate the overexpressed OXA-51-like enzyme in the OMP fraction (**Table 1**).

### Surface Motility Was Dramatically Reduced in CRAb Isolates

**FIGURE 3:**
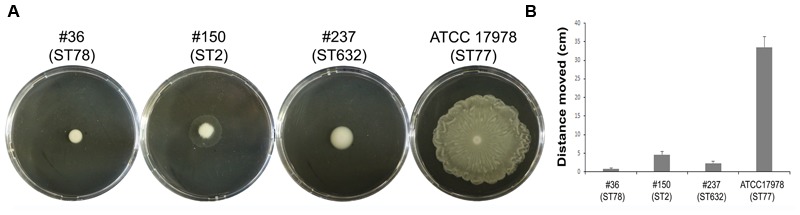
Motility of CRAb on agar plates. A representative picture of the motility pattern of each isolate is reported **(A)**. The distance migrated (diameter) for each strain was measured **(B)**; data are means ± standard deviation from at least three independent experiments, in duplicate (*P*-value < 0.01).

Motility contributes to virulence in many pathogens (Josenhans and Suerbaum, 2002). Indeed, motility might allow the spread of bacteria to both abiotic and biotic surfaces. Therefore, the surface-associated motility of each isolate was assayed on semi-solid LB agar plates (0.25%). The hypermotile ATCC 17978 reference strain was included in the assay as positive control (Wong et al., 2017 and references therein). Isolate #237 (ST632) showed a dramatically reduced surface motility with dense cells at the site of inoculation (**Figure 3**). Conversely, isolate #150 (ST2) displayed a motility pattern round with slightly jagged edges, and the cells were lighter in density (**Figure 3**). Isolate #36 (ST78) exhibited a non-motile phenotype. Results highlighted the overall low motility of our CRAb (**Figure 3**).

### All Isolates Showed a Weak Biofilm-Forming Phenotypes

**FIGURE 4:**
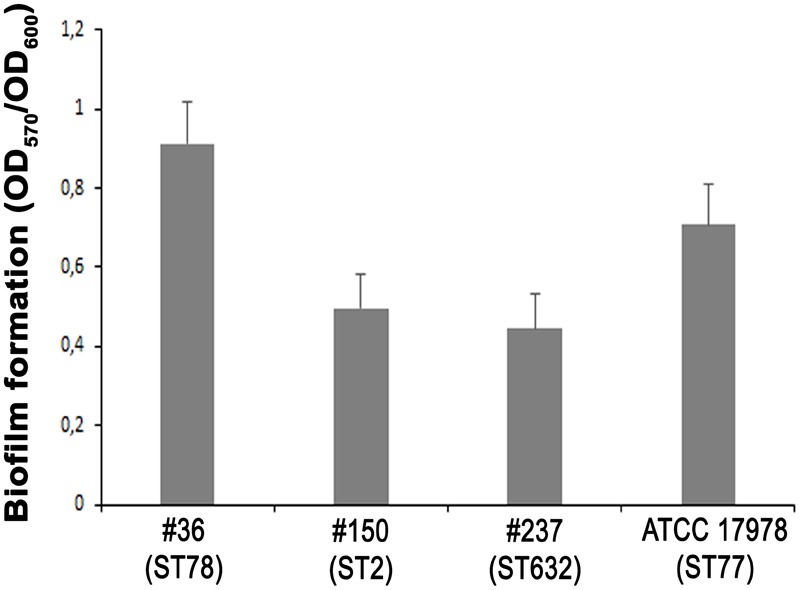
Biofilm formation of CRAb. Quantification of biofilm formation in *A. baumannii* clinical isolates. Data are means ± standard deviation from at least three independent experiments, eight wells per strain (*P*-value < 0.0001).

The ability of *A. baumannii* clinical isolates to adhere and form biofilms represent crucial features in host-pathogen interactions and in medical device-associated infections. Therefore, the biofilm-forming ability on polystyrene microtiter plates of each isolate was measured by Crystal Violet staining. Interestingly, isolate #36 (ST78) was capable to form significantly higher biofilm levels than the other isolates and ATCC 17978 reference strain (*P* < 0.01) (**Figure 4**). In contrast, both isolates #237 (ST632) and #150 (ST2) exhibited the weakest capacity to form biofilm (**Figure 4**). Therefore, all strains tested were able to form biofilm, although to a weak extent.

### ST78 and ST2 Were Able to Adhere to, Invade, and Survive within Epithelial Cells

**FIGURE 5:**
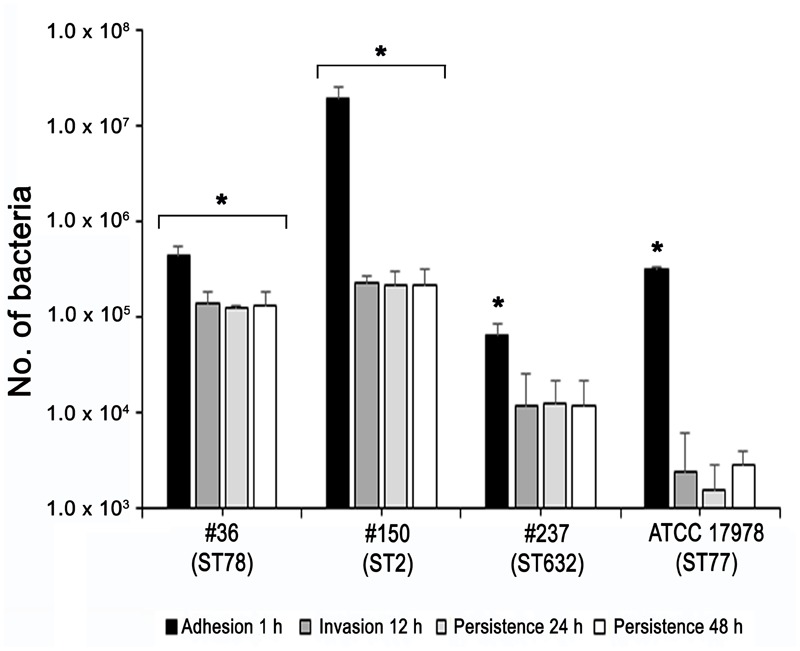
Ability of CRAb to adhere to, be internalized, and survive within cultured epithelial cells. Semi-confluent A549 cell monolayers were infected with each isolate or ATCC 17978 reference strain using a multiplicity of infection (MOI) of 100. Cell-surface-adherent bacteria were enumerated after 1-h incubation, whereas intracellular bacteria were counted after additional 12, 24, and 48-h of incubation in the presence of colistin (5 μg/ml). Data (CFU/ml) are means ± standard deviation from at least three independent experiments, in duplicate. Asterisks (^∗^) above the bars indicates a statistically significant difference among isolates (*P*-values < 0.01). Uninfected cells were used as control.

**FIGURE 6:**
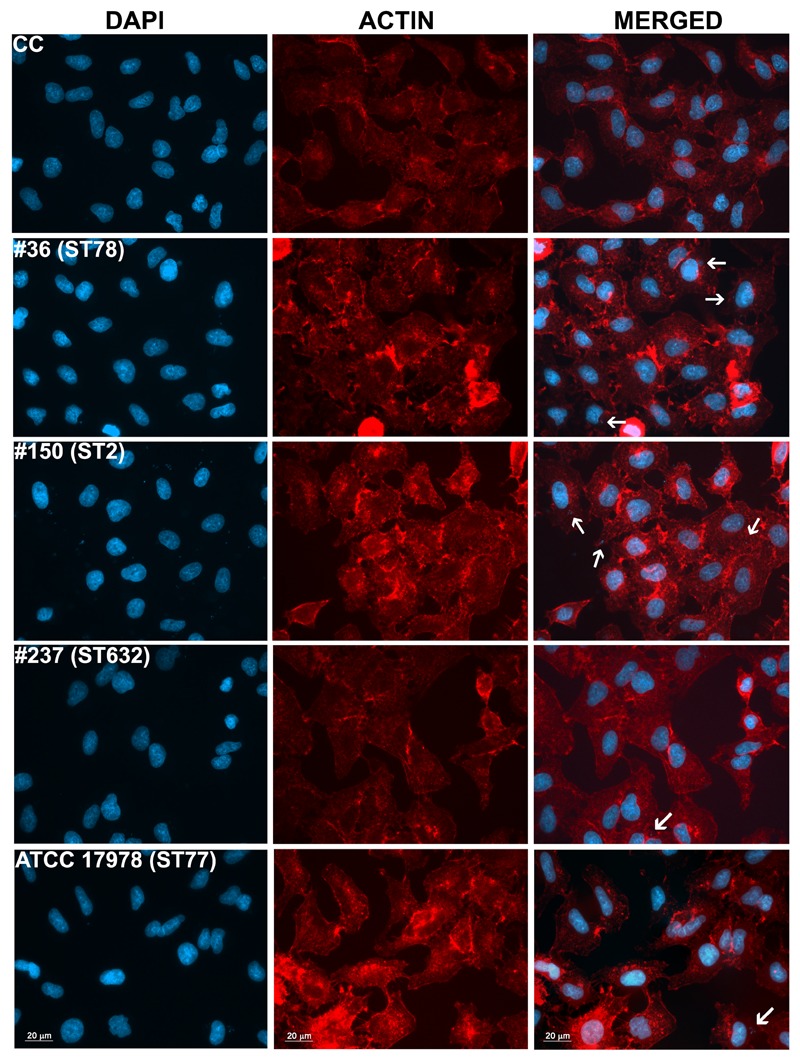
Intracellular localization of *A. baumannii* in epithelial cells. Semi-confluent A549 cell monolayers were infected as reported before. At 12-h post-infection, cells were fixed and actin was stained with rhodamine-conjugated phalloidin (red), while nuclei and bacterial DNA were stained with DAPI (blue). Images, representatives of at least three independent experiments, in duplicate, were captured using a Leica camera and processed using Qwin software (Leica). Arrows point representative intracellular bacteria. Uninfected cells were used as control (CC). Scale bar, 20 μm.

Adherence of bacteria to epithelial cells is an essential step to initiate infectious diseases. Therefore, the *in vitro* adherence of *A. baumannii* isolates to human lung epithelial cells was evaluated. A549 cell monolayers were infected with each isolates for 1 h and the number of cell-associated CFU was calculated (**Figure 5**). Isolate #150 (ST2) displayed a remarkable ability to adhere to epithelial cells, with a total number of adherent bacteria of 1.9 × 10^7^ ± 0.4 × 10^7^ CFU/ml (**Figure 5**). A lower amount of adherent bacteria were recovered for isolate #36 (ST78), 4,5 × 10^5^ ± 0.9 × 10^5^ CFU/ml (**Figure 5**). In contrast, cell adherence for isolate #237 (ST632) was very poor (6.4 × 10^4^ ± 2.0 × 10^4^ CFU/ml), even less to that measured for the ATCC 17978 reference strain (3.1 × 10^5^ ± 0.3 × 10^5^ CFU/ml) (**Figure 5**). It has been recently reported that some *A. baumannii* strains carry the FhaB/FhaC TPS system that is involved in an exceptional ability to adhere to A549 epithelial cells (Pérez et al., 2016). Due to its remarkable ability to adhere to cells and plate coating, we hypothesized that isolate #150 (ST2) could contain the genes encoding the FhaB/FhaC TPS system. Therefore, the presence of the *fhaC* gene was analyzed by PCR with specific primers designed to amplify the gene and its own promoter. Surprisingly, amplicons of the expected size were detectable only from the genomic DNA of isolate #36 (ST78). Sequence analysis confirmed the presence of an open reading frame of 1,758 bp matching the sequence (99% homology) of the *fhaC* gene from *A. baumannii* strain AbH12O-A2 (Pérez et al., 2016). No PCR products were obtained using genomic DNA from the ATCC 17978 reference strain, in agreement with a BLASTN search of its available genome.^4^ Next, to investigate whether CRAb were able to invade epithelial cells, a standard antibiotic protection assay using colistin was performed. The number of intracellular bacteria was assessed after 12 h of infection (**Figure 5**). Both isolates #36 (ST78) and #150 (ST2) were able to invade host cells, with a total number of intracellular bacteria of 1.4 × 10^5^ ± 0.4 × 10^5^ and 2.3 × 10^5^ ± 0.4 × 10^5^ CFU/ml, respectively (**Figure 5**). In line with the low adhesion rate, isolate #237 (ST632) was not invasive (1.2 × 10^4^ ± 1.3 × 10^4^ CFU/ml), as the ATCC 17978 reference strain (2.3 ± 3.1 CFU/ml) (**Figure 5**). Invasion results for the ATCC 17978 reference strain were in agreement with previous report (Giannouli et al., 2013). At the same time point of infection, the intracellular localization of either isolates #36 (ST78) or #150 (ST2) was qualitatively evaluated by fluorescence microscopy, following phalloidin and DAPI staining (**Figure 6**). To investigate whether *A. baumannii* isolates might survive and/or replicate intracellularly, the number of intracellular bacteria was monitored at 24 and 48 h after infection (**Figure 5**). Interestingly, the number of bacteria within host cells remained constant over time, highlighting the ability of both #36 (ST78) and #150 (ST2) isolates to survive within cells without intracellular replication (**Figure 5**). Noteworthy, even at 48 h after infection, monolayers remained intact and infected cells showed cell morphology and nuclear shape indistinguishable to uninfected cells (data not shown).

## Discussion

This study was aimed at identifying important phenotypic and molecular virulence traits of a new and common STs belonging to different ICs. Besides sharing specific features, data showed that both ST2 and ST78 genotypes have much more virulence-associated phenotypes than the ST632 genotype, in accordance to their higher infective rates in ICU patients, as previous reported (Ambrosi et al., 2016).

The structural composition of microbial cell surfaces can deeply influence the virulence of different isolates. As a first approach to analyze the surface components of different ST genotypes, we compared the OMP profiles among CRAb. OmpA was present and highly expressed in all strains analyzed, irrespective of their antibiotype (**Figure 1**). This pleiotropic β-barrel porin is widely conserved among Gram-negative bacteria. In *A. baumannii*, OmpA was shown to be involved in the interaction with epithelial cells, biofilm-forming activity, antibiotic resistance, and cell death (Choi et al., 2008a,b; Gaddy et al., 2009; Smani et al., 2014). However, in our experimental conditions and despite OmpA overexpression, monolayers remained intact and cell morphology and nuclear shape of cells infected with either #36 (ST78) or #150 (ST2) isolates were indistinguishable to uninfected cells, even at 48 h post-infection (data not shown). As other respiratory pathogens, *A. baumannii* cells might display the small molecule phosphorylcholine on PorinD (Smani et al., 2012). Phosphorylcholine, by mimicking the chemokine platelet activator factor (PAF), mediates the binding to the human platelet activating factor receptor (PAFr), triggering bacterial internalization into the host cells (Smani et al., 2012). Indeed, we found that all *A. baumannii* displaying phosphorylcholine-porinD adhered to human lung epithelial cells (**Figure 5**). However, not all strains were subsequently able to invade the cells. Comparison of the OMP profiles among CRAb highlighted their overall heterogeneity, particularly with respect to that of the ATCC 17978 reference strain. There are contrasting data in the literature on the changes of the OMP profiles in CRAb (Limansky et al., 2002; del Mar Tomás et al., 2005; Zander et al., 2013; Cardoso et al., 2016). This apparent discrepancy could be related to the mechanism evolved/acquired for antibiotic resistance by each strain. Dramatic changes in the OMP profile were reported for CRAb with non-enzymatic carbapenem-resistance mechanisms, due to a reduction in the expression of OMPs (Nowak and Paluchowska, 2016). Interestingly, the Omp33–36 kDa porin was strongly expressed in all isolates, especially in isolate #150 (ST2) (Supplementary Table S2). Conversely, CarO was found to be significantly expressed in all CRAb whereas undetectable in the ATCC 17978 reference strain (**Table 1**). Herein, we show that there is a slight modulation in the expression of Omp33–36 kDa porin, whereas it confirms that overexpression of CarO is a common feature of CRAb (Cardoso et al., 2016) Our result reinforces previous findings that CarO represents the specific OM channel for carbapenem influx in CRAb overexpressing OXA enzymes (Mussi et al., 2007; Morán-Barrio et al., 2017). CRAb used in this study carry OXA-51 or both OXA-51 and OXA-23 (Ambrosi et al., 2016). Remarkably, we found overexpressed OXA β-lactamases belonging to the OXA-23-like and/or OXA-51-like groups in the OMP fraction of CRAb, whereas no overexpressed OXA-51-like enzyme could be detected in the OMP fraction from ATCC 17978 reference strain (**Table 1**). Interestingly, co-purification of OXA-23 enzymes within the OMP fraction with or without the use of a cross-linking agent was previously reported (Fajardo Bonin et al., 2014; Wu et al., 2016). Wu et al. (2016) elegantly demonstrated that the periplasmic OXA-23 enzyme was mainly detectable from OM-enriched fractions of AB5075 strain and interacted *in vivo* with several OMPs, including OmpA, CarO, YiaD, OmpW, Omp 33–36, and AdeC/K. This complex network of interactions between OMPs and the OXA-23 enzyme was shown to drive its localization mainly at the inner surface of the OM (Wu et al., 2016). Herein, we demonstrate that also an overexpressed OXA-51-like enzyme co-purifies with the OMP fraction even in the absence of any cross-linker agent (**Table 1**). Therefore, detection of both overexpressed OXA-23 and OXA-51 enzymes and the absence of the OXA-51 enzyme from the carbapenem-susceptible ATCC 17978 reference strain within the OMP fraction corroborates and extends previous results about their interaction and localization, suggesting for the first time a common behavior in OXA-expressing CRAb. On this basis, we believe that each OXA enzyme might interact and co-localize with selected OMPs, mainly CarO in our strains, which act as anchors to concentrate OXA right below their channel entrance, thereby conferring the best protection against carbapenems. Conversely, it is possible that metalloenzymes require a different OMP profile, being the mechanism of carbapenem hydrolysis substantially different from OXA enzymes. Biofilms and motility are important and interconnected capacities that enable bacteria to persist in the environment and colonize the host. In some bacteria, motility has been associated to increased biofilm production and virulence [i.e., *Listeria monocytogenes, Pseudomonas aeruginosa*, and *Burkholderia cepacia* (Harshey, 2003)]. All our CRAb showed poor ability to form biofilms and to move (**Figures 3, 4**); however, the number of strains analyzed in this study is too little to lead to any conclusion.

Since the majority of *A. baumannii* infections affects the pulmonary system (Wong et al., 2017), the epithelial A549 cell line was chosen as a model system for investigating adherence to, invasion, and survival of CRAb *in vitro*. The total number of adherent bacteria ranged between 6.4 × 10^4^ ± 2.0 × 10^4^ and 4.5 × 10^5^ ± 0.9 × 10^5^ CFU/ml for all the strains analyzed except for isolate #150 (ST2) for which the total number of adherent bacteria was 1.9 × 10^7^ ± 0.4 × 10^7^ CFU/ml (**Figure 5**). This remarkable adhesiveness led us to investigate the presence of the recently discovered AbFhaB/AbFhaC TPS system (Pérez et al., 2016), which we found only in the isolate #36 (ST78). In particular, the AbFhaB of the TPS system was shown to mediate strong adherence to epithelial cells via the host cell protein fibronectin (Pérez et al., 2016). Indeed, other bacterial proteins were shown to interact with fibronectin in *A. baumannii*, such as OmpA, Omp33-36, EF-Tu, and TonB proteins (Mortensen and Skaar, 2013), all proteins identified in our CRAb (Supplementary Table S2). Therefore, the remarkable adhesiveness of isolate #150 (ST2) to epithelial cells and to cell culture dishes might be related to the autotransporter protein Ata that mediates adhesion to type IV collagen and/or to specific bacterial appendages, unrelated to those involved in biofilm formation, being this isolate a poor biofilm-producer (**Figures 4, 5**). Indeed, type IV pili are involved in a wide range of bacterial processes including motility and host-cell adhesion and were shown to be highly variable in *A. baumannii* belonging to IC 2 (Harshey, 2003; Piepenbrink et al., 2016). Hence, these extracellular adhesive appendages might be involved in the noticeable adhesion of isolate #150 (ST2) to A549 cells as well as to rough surfaces such as those of cell culture dishes. In support of this possibility, isolate #150 (ST2) was shown to have a partial motile phenotype (**Figure 3**).

Despite the differential extent of bacterial adhesion, the 31.3% of total adherent bacteria of isolate #36 (ST78) were internalized by 12 h, only the 1.2% in the case of isolate #150 (ST2), whereas cellular invasion of isolate #237 (ST632) was almost undetectable (**Figure 5**). In characterizing *A. baumannii* invasion further, we observed that both invasive isolates were capable of intracellular survival for up to 48 h, without causing apparent cytotoxic effects in A549 cells (**Figure 5**). Interestingly, both isolates shared the highest number of proteins identified in this study (56 proteins, 32%) with respect to isolate #237 (ST632) and ATCC 17978 reference strain (**Figure 2** and Supplementary Table S2). Indeed, it has been previously shown that adherent bacteria enter non-phagocytic cells via a zipper mechanism mediated by the interaction between the phosphorylcholine-porinD and PAFr (Smani et al., 2012). PAFr engagement promotes the binding of β-arrestins to activated and phosphorylated receptors of G proteins and clathrin that, together with the reorganization of host cell actin filament and microtubule, allow bacterial endocytosis (Smani et al., 2012). Noteworthy, several major human pathogens of the respiratory tract, such as *Streptococcus pneumoniae, Neisseria meningitidis, Haemophilus influenzae*, and *P. aeruginosa*, carry surface exposed structures/proteins modified with phosphorylcholine to allow binding of PAFr and activate the endocytosis machinery cascade (Clark and Weiser, 2013). However, despite detection of phosphorylcholine-porinD, cellular invasion of isolate #237 (ST632) was not detected under our experimental conditions (**Figure 5**). Therefore, it is reasonable to believe that modification of porinD is necessary to bind PAFr but not sufficient for the internalization of isolate #237 (ST632) and additional factor(s) are required for clathrin-mediated endocytosis. Conversely, data reported in this study indicate that the firm attachment to A549 cells and, possibly, the expression of additional factor(s) confer to isolate #36 (ST78) a higher ability to invade the host with respect to other isolates. The intracellular survival of both isolates #36 (ST78) and #150 (ST2) and the lack of apparent cytotoxic effects in A549 cells was unexpected (**Figure 5**). Indeed, Smani et al. (2011) showed cell death in *in vitro* experiment 24 h post-infection using a clinical outbreak pan-resistant strain and the susceptible ATCC 19606 reference strain. Conversely, data presented by Lee et al. (2001) led to the conclusion that secreted factors, rather than surface proteins or structures were responsible for the very early apoptotic cell death. Noteworthy, 157 genes were identified as necessary for survival in the ATCC 17978 reference strain using a murine model of pneumonia (Wang et al., 2014). Surprisingly, only few genes were found to be related to virulence traits, whereas the majority of the identified genes were involved in bacterial metabolism and transport (Wang et al., 2014). Therefore, it can be concluded that the ability to survive within host cells or induce cell death might be related to few but specific features of the strain analyzed. Indeed, despite the high variability in surface exposed structures reflecting the different phenotypes observed for each isolate, shared OMPs might allow invasion and survival within host cells. Finally, the fate of *A. baumannii* intracellular survival after 48 h and its biological meaning are still unknown. The study of *A. baumannii* intracellular life cycle and intracellular trafficking will improve our knowledge on key virulence determinants, thereby enabling the development of effective therapeutics against this emerging threat.

## Author Contributions

CA and DS conceived and designed the work. CA, DS, MA, LDF, and LP performed the experiments. CA, DS, and LDF analyzed the data. CZ contributed to reagents/materials/analysis tools. CA, DS, and ATP wrote the manuscript. All authors read and approved the final manuscript.

## Conflict of Interest Statement

The authors declare that the research was conducted in the absence of any commercial or financial relationships that could be construed as a potential conflict of interest. The handling Editor declared a shared department, though no other collaboration, with one of the authors, DS, and states that the process nevertheless met the standards of a fair and objective review.

## Footnotes

1 www.uniprot.org

2 http://www.psort.org

3 http://bioinfogp.cnb.csic.es/tools/venny/index.html

4 https://www.ncbi.nlm.nih.gov

## References

[B1] Ambrosi C. Aleandri M. Giordano A. Scribano D. Marazzato M. Zagaglia C. (2016). Molecular characterisation of extensively drug-resistant *Acinetobacter baumannii*: first report of a new sequence type in Italy. *J. Glob. Antimicrob. Resist.* 7 154–156. 10.1016/j.jgar.2016.10.002 27835843

[B2] Beceiro A. Tomás M. Bou G. (2013). Antimicrobial resistance and virulence: a successful or deleterious association in the bacterial world? *Clin. Microbiol. Rev.* 26 185–230. 10.1128/CMR.00059-12 23554414 PMC3623377

[B3] Cardoso J. P. Cayô R. Girardello R. Gales A. C. (2016). Diversity of mechanisms conferring resistance to β-lactams among OXA-23-producing *Acinetobacter baumannii* clones. *Diagn. Microbiol. Infect. Dis.* 85 90–97. 10.1016/j.diagmicrobio.2016.01.018 26971181

[B4] Choi C. H. Hyun S. H. Lee J. Y. Lee J. S. Lee Y. S. Kim S. A. (2008a). *Acinetobacter baumannii* outer membrane protein A targets the nucleus and induces cytotoxicity. *Cell Microbiol.* 10 309–319. 17760880 10.1111/j.1462-5822.2007.01041.x

[B5] Choi C. H. Lee J. S. Lee Y. C. Park T. I. Lee J. C. (2008b). *Acinetobacter baumannii* invades epithelial cells and outer membrane protein A mediates interactions with epithelial cells. *BMC Microbiol.* 8:216. 10.1186/1471-2180-8-216 19068136 PMC2615016

[B6] Clark S. E. Weiser J. N. (2013). Microbial modulation of host immunity with the small molecule phosphorylcholine. *Infect. Immun.* 81 392–401. 10.1128/IAI.01168-12 23230294 PMC3553803

[B7] Clemmer K. M. Bonomo R. A. Rather P. N. (2011). Genetic analysis of surface motility in *Acinetobacter baumannii*. *Microbiology* 157 2534–2544. 10.1099/mic.0.049791-0 21700662 PMC3352170

[B8] Cuenca F. F. Pascual A. Martínez Marínez L. Conejo M. C. Perea E. J. (2003). Evaluation of SDS-polyacrylamide gel systems for the study of outer membrane protein profiles of clinical strains of *Acinetobacter baumannii*. *J. Basic Microbiol.* 43 194–201. 10.1002/jobm.200390022 12761770

[B9] del Mar Tomás M. Beceiro A. Pérez A. Velasco D. Moure R. Villanueva R. (2005). Cloning and functional analysis of the gene encoding the 33- to 36-kilodalton outer membrane protein associated with carbapenem resistance in *Acinetobacter baumannii*. *Antimicrob. Agents Chemother.* 49 5172–5175. 10.1128/AAC.49.12.5172-5175.2005 16304197 PMC1315955

[B10] Di Francesco L. Correani V. Fabrizi C. Fumagalli L. Mazzanti M. Maras B. (2012). 14-3-3𝜀 marks the amyloid stimulated microglia long-term activation. *Proteomics* 12 124–134. 10.1002/pmic.201100113 22065591

[B11] Diancourt L. Passet V. Nemec A. Dijkshoorn L. Brisse S. (2010). The population structure of *Acinetobacter baumannii*: expanding multiresistant clones from an ancestral susceptible genetic pool. *PLOS ONE* 5:e10034. 10.1371/journal.pone.0010034 20383326 PMC2850921

[B12] Fajardo Bonin R. Chapeaurouge A. Perales J. da Silva J. G. Jr. do Nascimento H. J. D’Alincourt Carvalho Assef A. P. (2014). Identification of immunogenic proteins of the bacterium *Acinetobacter baumannii* using a proteomic approach. *Proteomics Clin. Appl.* 8 916–923. 10.1002/prca.201300133 24899143

[B13] Gaddy J. A. Tomaras A. P. Actis L. A. (2009). The *Acinetobacter baumannii* 19606 OmpA protein plays a role in biofilm formation on abiotic surfaces and in the interaction of this pathogen with eukaryotic cells. *Infect. Immun.* 77 3150–3160. 10.1128/IAI.00096-09 19470746 PMC2715673

[B14] Giannouli M. Antunes L. C. Marchetti V. Triassi M. Visca P. Zarrilli R. (2013). Virulence-related traits of epidemic *Acinetobacter baumannii* strains belonging to the international clonal lineages I-III and to the emerging genotypes ST25 and ST78. *BMC Infect. Dis.* 13:282. 10.1186/1471-2334-13-282 23786621 PMC3691691

[B15] Giannouli M. Cuccurullo S. Crivaro V. Di Popolo A. Bernardo M. Tomasone F. (2010). Molecular epidemiology of multidrug-resistant *Acinetobacter baumannii* in a tertiary care hospital in Naples, Italy, shows the emergence of a novel epidemic clone. *J. Clin. Microbiol.* 48 1223–1230. 10.1128/JCM.02263-09 20181918 PMC2849555

[B16] Harshey R. M. (2003). Bacterial motility on a surface: many ways to a common goal. *Annu. Rev. Microbiol.* 57 249–273. 10.1146/annurev.micro.57.030502.091014 14527279

[B17] Josenhans C. Suerbaum S. (2002). The role of motility as a virulence factor in bacteria. *Int. J. Med. Microbiol.* 291 605–614. 10.1078/1438-4221-00173 12008914

[B18] Karah N. Sundsfjord A. Towner K. Samuelsen O. (2012). Insights into the global molecular epidemiology of carbapenem non-susceptible clones of *Acinetobacter baumannii*. *Drug Resist. Updat.* 15 237–247. 10.1016/j.drup.2012.06.001 22841809

[B19] Laemmli U. K. (1970). Cleavage of structural proteins during the assembly of the head of bacteriophage T4. *Nature* 277 680–685. 10.1038/227680a0 5432063

[B20] Lee J. C. Oh J. Y. Kim K. S. Jeong Y. W. Park J. C. Cho J. W. (2001). Apoptotic cell death induced by *Acinetobacter baumannii* in epithelial cells through caspase-3 activation. *APMIS* 109 679–684. 10.1034/j.1600-0463.2001.d01-132.x 11890571

[B21] Limansky A. S. Mussi M. A. Viale A. M. (2002). Loss of a 29-kilodalton outer membrane protein in *Acinetobacter baumannii* is associated with imipenem resistance. *J. Clin. Microbiol.* 40 4776–4778. 10.1089/mdr.2009.0828 12454194 PMC154632

[B22] Morán-Barrio J. Cameranesi M. M. Relling V. Limansky A. S. Brambilla L. Viale A. M. (2017). The acinetobacter outer membrane contains multiple specific channels for carbapenem β-lactams as revealed by kinetic characterization analyses of imipenem permeation into *Acinetobacter baylyi* cells. *Antimicrob. Agents Chemother.* 61:e01737-16. 10.1128/AAC.01737-16 28069648 PMC5328561

[B23] Mortensen B. L. Skaar E. P. (2013). The contribution of nutrient metal acquisition and metabolism to *Acinetobacter baumannii* survival within the host. *Front. Cell. Infect. Microbiol.* 3:95. 10.3389/fcimb.2013.00095 24377089 PMC3859900

[B24] Mussi M. A. Relling V. M. Limansky A. S. Viale A. M. (2007). CarO, an *Acinetobacter baumannii* outer membrane protein involved in carbapenem resistance, is essential for L-ornithine uptake. *FEBS Lett.* 581 5573–5578. 10.1016/j.febslet.2007.10.063 17997983

[B25] Nowak P. Paluchowska P. (2016). *Acinetobacter baumannii*: biology and drug resistance - role of carbapenemases. *Folia Histochem. Cytobiol.* 54 61–74. 10.5603/FHC.a2016.0009 27270503

[B26] Pérez A. Merino M. Rumbo-Feal S. Álvarez-Fraga L. Vallejo J. A. Beceiro A. (2016). The FhaB/FhaC two-partner secretion system is involved in adhesion of *Acinetobacter baumannii* AbH12O-A2 strain. *Virulence* 18 1–16. 10.1080/21505594.2016.1262313 27858524 PMC5626241

[B27] Piepenbrink K. H. Lillehoj E. Harding C. M. Labonte J. W. Zuo X. Rapp C. A. (2016). Structural Diversity in the Type IV Pili of Multidrug-resistant Acinetobacter. *J. Biol. Chem.* 291 22924–22935. 10.1074/jbc.M116.751099 27634041 PMC5087714

[B28] Potron A. Poirel L. Nordmann P. (2015). Emerging broad-spectrum resistance in *Pseudomonas aeruginosa* and *Acinetobacter baumannii*: Mechanisms and epidemiology. *Int. J. Antimicrob. Agents.* 45 568–585. 10.1016/j.ijantimicag.2015.03.001 25857949

[B29] Principe L. Piazza A. Giani T. Bracco S. Caltagirone M. S. Arena F. (2014). Epidemic diffusion of OXA-23-producing *Acinetobacter baumannii* isolates in Italy: results of the first cross-sectional countrywide survey. *J. Clin. Microbiol.* 52 3004–3010. 10.1128/JCM.00291-14 24920776 PMC4136168

[B30] Schweppe D. K. Harding C. Chavez J. D. Wu X. Ramage E. Singh P. K. (2015). Host-Microbe Protein Interactions during Bacterial Infection. *Chem. Biol.* 22 1521–1530. 10.1016/j.chembiol.2015.09.015 26548613 PMC4756654

[B31] Smani Y. Docobo-Pérez F. López-Rojas R. Domínguez-Herrera J. Ibáñez-Martínez J. Pachón J. (2012). Platelet-activating factor receptor initiates contact of *Acinetobacter baumannii* expressing phosphorylcholine with host cells. *J. Biol. Chem.* 287 26901–26910. 10.1074/jbc.M112.344556 22689572 PMC3411026

[B32] Smani Y. Docobo-Pérez F. McConnell M. J. Pachón J. (2011). *Acinetobacter baumannii*-induced lung cell death: role of inflammation, oxidative stress and cytosolic calcium. *Microb. Pathog.* 50 224–232. 10.1016/j.micpath.2011.01.008 21288481

[B33] Smani Y. Dominguez-Herrera J. Pachón J. (2013). Association of the outer membrane protein Omp33 with fitness and virulence of *Acinetobacter baumannii*. *J. Infect. Dis.* 208 1561–1570. 10.1093/infdis/jit386 23908480

[B34] Smani Y. Fàbrega A. Roca I. Sánchez-Encinales V. Vila J. Pachón J. (2014). Role of OmpA in the multidrug resistance phenotype of *Acinetobacter baumannii*. *Antimicrob. Agents Chemother.* 58 1806–1808. 10.1128/AAC.02101-13 24379205 PMC3957889

[B35] Smani Y. Pachón J. (2013). Loss of the OprD homologue protein in *Acinetobacter baumannii*: impact on carbapenem susceptibility. *Antimicrob. Agents Chemother.* 57:677. 10.1128/AAC.01277-12 23275492 PMC3535904

[B36] Stepanović S. Vuković D. Hola V. Di Bonaventura G. Djukić S. Cirković I. (2007). Quantification of biofilm in microtiter plates: overview of testing conditions and practical recommendations for assessment of biofilm production by staphylococci. *APMIS* 115 891–899. 10.1111/j.1600-0463.2007.apm_630.x 17696944

[B37] Thein M. Sauer G. Paramasivam N. Grin I. Linke D. (2010). Efficient subfractionation of gram-negative bacteria for proteomics studies. *J. Proteome Res.* 9 6135–6147. 10.1021/pr1002438 20932056

[B38] Vijayakumar S. Rajenderan S. Laishram S. Anandan S. Balaji V. Biswas I. (2016). Biofilm formation and motility depend on the nature of the *Acinetobacter baumannii* clinical isolates. *Front. Public Health* 4:105. 10.3389/fpubh.2016.00105 27252939 PMC4877508

[B39] Wang N. Ozer E. A. Mandel M. J. Hauser A. R. (2014). Genome-wide identification of *Acinetobacter baumannii* genes necessary for persistence in the lung. *MBio* 5 e1163–e1114. 10.1128/mBio.01163-14 24895306 PMC4049102

[B40] Weber B. S. Harding C. M. Feldman M. F. (2015). Pathogenic Acinetobacter: from the cell surface to infinity and beyond. *J. Bacteriol.* 198 880–887. 10.1093/infdis/jit386 26712938 PMC4772598

[B41] Wong D. Nielsen T. B. Bonomo R. A. Pantapalangkoor P. Luna B. Spellberg B. (2017). Clinical and Pathophysiological Overview of Acinetobacter Infections: a Century of Challenges. *Clin. Microbiol. Rev.* 30 409–447. 10.1128/CMR.00058-16 27974412 PMC5217799

[B42] Wu X. Chavez J. D. Schweppe D. K. Zheng C. Weisbrod C. R. Eng J. K. (2016). In vivo protein interaction network analysis reveals porin-localized antibiotic inactivation in *Acinetobacter baumannii* strain AB5075. *Nat. Commun.* 7:13414. 10.1038/ncomms13414 27834373 PMC5114622

[B43] Zander E. Chmielarczyk A. Heczko P. Seifert H. Higgins P. G. (2013). Conversion of OXA-66 into OXA-82 in clinical *Acinetobacter baumannii* isolates and association with altered carbapenem susceptibility. *J. Antimicrob. Chemother.* 68 308–311. 10.1093/jac/dks382 23014718

[B44] Zarrilli R. Pournaras S. Giannouli M. Tsakris A. (2013). Global evolution of multidrug-resistant *Acinetobacter baumannii* clonal lineages. *Int. J. Antimicrob. Agents* 41 11–19. 10.1016/j.ijantimicag.2012.09.008 23127486

