# Supplementary material for: Acinetobacter baumannii Virulence Traits: A Comparative Study of a Novel Sequence Type with Other Italian Endemic International Clones
Source: Front Microbiol. 2017 Oct 12;8:1977. doi: 10.3389/fmicb.2017.01977 (PMC5643476; doi:10.3389/fmicb.2017.01977)
Supplement: Supplementary file 1 [file Table_1.docx]

Supplementary Material

***Acinetobacter baumannii* virulence traits: a comparative study of a novel sequence type with other Italian endemic international clones**

Cecilia Ambrosi^*^, Daniela Scribano, Marta Aleandri, Carlo Zagaglia, Laura Di Francesco, Lorenza Putignani, Anna Teresa Palamara

*** Correspondence:**

Cecilia Ambrosi

[cecilia.ambrosi@uniroma1.it](mailto:cecilia.ambrosi@uniroma1.it)

Table S1. Main characteristics of *A. baumannii* strains used in this study.

| Strain | Relevant characteristics | | | Reference or source |
| --- | --- | --- | --- | --- |
|  | Sequence Type (ST) | *bla*_OXA_ gene | Antibiotic Susceptibility* |  |
| #36 | 78 | *bla*_OXA-51-like_ | AK^S^, CST^S^, TGC^S^ | Ambrosi et al., 2016 |
| #150 | 2 | *bla*_OXA-51-like_  *bla*_OXA-23-like_ | CST^S^ | Ambrosi et al., 2016 |
| #237 | 632 | *bla*_OXA-51-like_  *bla*_OXA23-like_ | CST^S^, TGC^S^ | Ambrosi et al., 2016 |
| ATCC 17978 | 77 | *bla*_OXA-51-like_ |  | Reference strain |

*Antibiotic tested: AK, Amikacin; GM, Gentamicin; A/AA, Amoxicillin/clavulanic acid; AMP, Ampicillin; P/T, Piperacillin/Tazobactam; CPE, Cefepime; CTX, Cefotaxime; CAZ, Ceftazidime; CIP, Ciprofloxacin; T/S, Trimethoprim/Sulfamethoxazole; IMP, Imipenem; CST, Colistin; TGC, Tigecycline.
